# ESR1 ChIP-Seq Identifies Distinct Ligand-Free ESR1 Genomic Binding Sites in Human Hepatocytes and Liver Tissue

**DOI:** 10.3390/ijms22031461

**Published:** 2021-02-02

**Authors:** Joseph M. Collins, Zhiguang Huo, Danxin Wang

**Affiliations:** 1 Center for Pharmacogenomics, Department of Pharmacotherapy and Translational Research, College of Pharmacy, University of Florida, Gainesville, FL 32610, USA; jcoll86@ufl.edu; 2 Department of Biostatistics, College of Public Health & Health Professions, University of Florida, Gainesville, FL 32610, USA; zhuo@ufl.edu

**Keywords:** estrogen receptor alpha, ESR1, ChIP-Seq, genomic binding, cytochrome p450s

## Abstract

The estrogen receptor alpha (ESR1) is an important gene transcriptional regulator, known to mediate the effects of estrogen. Canonically, ESR1 is activated by its ligand estrogen. However, the role of unliganded ESR1 in transcriptional regulation has been gaining attention. We have recently shown that ligand-free ESR1 is a key regulator of several cytochrome P450 (CYP) genes in the liver, however ligand-free ESR1 has not been characterized genome-wide in the human liver. To address this, ESR1 ChIP-Seq was conducted in human liver samples and in hepatocytes with or without 17beta-estradiol (E2) treatment. We identified both ligand-dependent and ligand-independent binding sites throughout the genome. These two ESR1 binding categories showed different genomic localization, pathway enrichment, and cofactor colocalization, indicating different ESR1 regulatory function depending on ligand availability. By analyzing existing ESR1 data from additional human cell lines, we uncovered a potential ligand-independent ESR1 activity, namely its co-enrichment with the zinc finger protein 143 (ZNF143). Furthermore, we identified ESR1 binding sites near many gene loci related to drug therapy, including the CYPs. Overall, this study shows distinct ligand-free and ligand-bound ESR1 chromatin binding profiles in the liver and suggests the potential broad influence of ESR1 in drug metabolism and drug therapy.

## 1. Background

The estrogen receptor alpha (ERα, ESR1) is one of the best characterized nuclear receptors due to its extensive roles regulating physiology, pathophysiology, and development of diverse tissues (see reviews by [1,2,3]), in particular its role in breast cancer progression [4]. Besides reproductive tissues, ESR1 is known to have an important role in the liver, regulating lipid and glucose metabolism [5,6]. Altered ESR1 expression is associated with development of hepatocellular carcinomas [7,8], and is related to the sexually dimorphic nature of several liver diseases, for example, hepatocellular carcinomas [9], nonalcoholic fatty liver disease [6,10,11], and liver gene expression [12] including the cytochrome P450 (CYP) drug metabolizing enzyme CYP3A4 [13].

Like the other nuclear receptors, ESR1 canonically functions as a ligand-dependent transcription factor (TF) where association with estrogen promotes ESR1 binding to the estrogen response element (ERE) and thereby enables regulation of target genes. However, binding of ESR1 to DNA in the absence of estrogen has been shown at specific promoters in osteoblasts [14] and MCF-7 breast cancer cells [15]. By depleting ESR1 in MCF- 7 cells, Caizzi et al. showed that ligand-free ESR1 binds to several thousand locations throughout the genome and regulates the expression of hundreds of genes, indicating that ESR1 maintains regulatory function in the absence of estrogen [16]. More recently, a study thoroughly categorized ESR1 binding in MCF-7 cells, identifying distinct binding sites both before and after E2 treatment, further supporting estrogen-mediated and ligand-free ESR1 binding sites throughout the genome [17].

Recently, we identified ligand-free ESR1 as a master regulator for the expression of several cytochrome P450s (CYPs) including CYP3A4 [18]. CYP3A4 is the most abundant drug metabolizing enzyme in human liver and is responsible for metabolism of numerous drugs [19]. Up- or down-regulation of ESR1 in hepatocytes or the hepatic-derived cell line Huh7 changed the expression of multiple CYP enzymes [18], implicating the broad influence of ESR1 on the metabolism of xenobiotics. While estrogen-induced expression of several CYP enzymes (e.g., CYP2A6/CYP1A1) [20,21] is known, mechanisms underlying ligand-free ESR1 regulation in the liver remain uncertain.

To better understand the role of ESR1 in the liver, we conducted ESR1 Chromatin Immunoprecipitation followed by high throughput sequencing (ChIP-Seq) in human liver samples and in primary culture hepatocytes with or without 17-beta-estradiol (E2) treatment. Consistent with results in MCF-7 cells [16,17], we found distinct chromatin binding sites of ligand-bound and ligand-free ESR1. Our results indicate that ESR1 regulates key processes in the liver via ligand-dependent and ligand-independent roles. To our knowledge, this is the first ESR1 ChIP-Seq study conducted in human liver tissues and in hepatocytes.

## 2. Results

### 2.1. ESR1 Genomic Localization in Hepatocytes and the Liver Tissues

**Figure 1:**
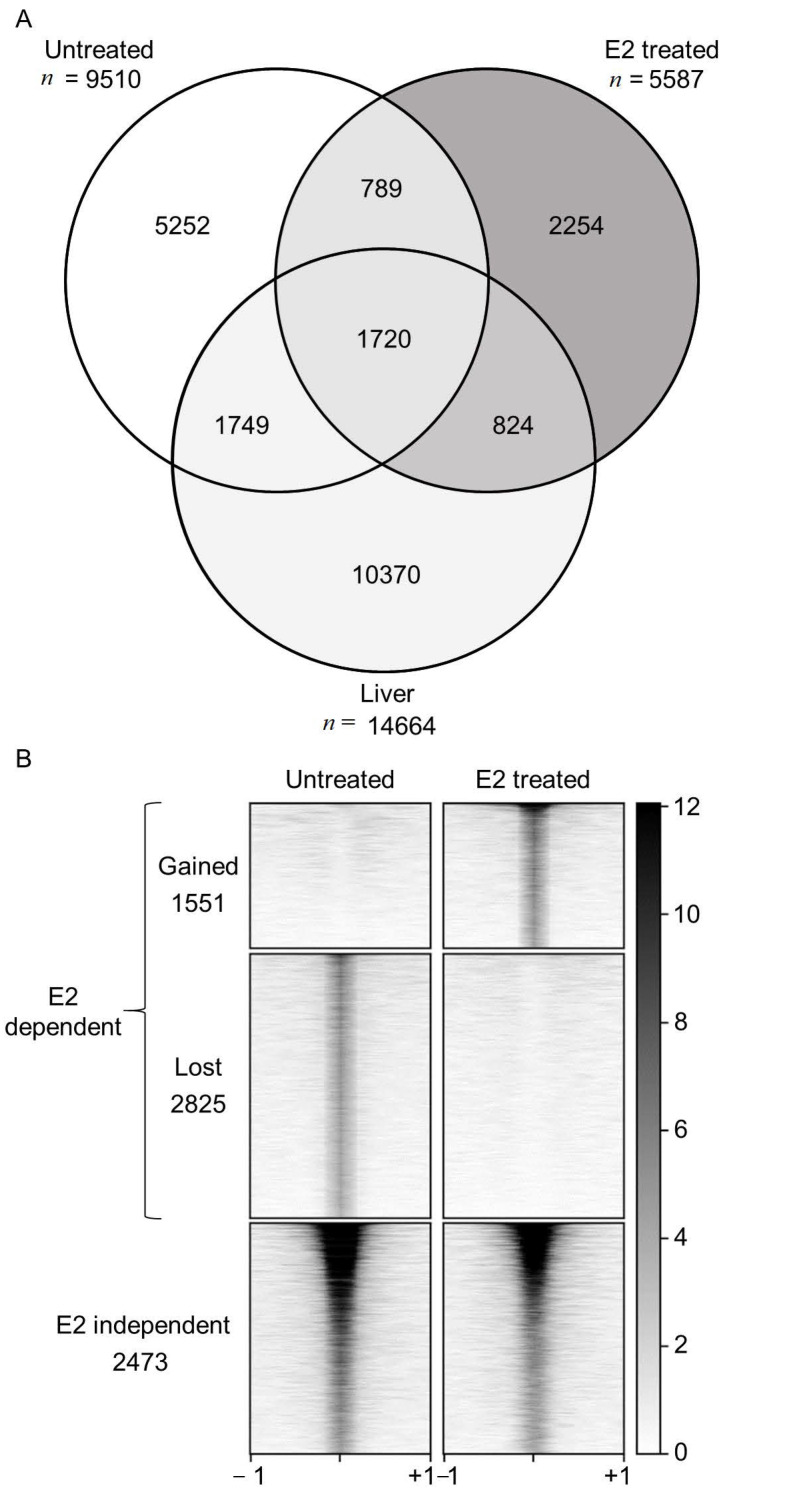
ESR1 ChIP-Seq peaks identified and classification of ESR1 binding in response to E2 treatment. (**A**) Venn diagram detailing peak overlap in each sample. (**B**) ChIP-seq read density heatmap showing ESR1 enrichment in each binding category. Density plots are centered on ESR1 peaks within a ± 1 kb window. Darker coloration indicates higher ChIP-Seq read counts.

We detail ESR1 binding in the liver genome-wide using ChIP-Seq. Six liver biopsy samples were combined (three males and three females, see Supplemental Table S1 for demographic information of donors) and chromatin immunoprecipitation was conducted against ESR1 and compared to an input DNA control. To investigate ESR1 chromatin localization changes in response to estrogen treatment, ESR1 ChIP-Seq was also conducted in primary culture hepatocytes grown in media lacking estrogen and treated with DMSO (untreated) or treated with E2 (E2-treated). Analysis of ESR1 enrichment in these samples identified ESR1 binding sites throughout the genome (number of peaks, liver *n* = 14,664, E2-treated *n* = 5587 and untreated *n* = 9510) (Figure 1A). Example ChIP-Seq peaks are shown in Supplemental Figure S1. Peaks were then assessed for any sequence overlap between samples, revealing 1720 shared ESR1 binding sites among all three samples. These 1720 peaks contributed to 18%, 31%, and 12% of peaks identified in untreated, E2- treated, and liver data sets, respectively.

Differential binding of ESR1 in response to E2 treatment was determined by comparing the E2-treated and untreated samples. ESR1 binding was then categorized into three groups. ESR1 binding sites identified in E2-treated hepatocytes that were at least four times higher than in untreated hepatocytes were labeled as “gained with treatment” (*n* = 1551). Conversely, peaks identified in untreated hepatocytes compared to E2-treated hepatocytes were considered “lost after treatment” (*n* = 2825). Finally, peaks that were found in both treated and untreated samples (i.e., peaks that are not different by 4-fold between these two conditions) were categorized as “E2-independent” (*n* = 2473) (Figure 1B). The 1720 peaks that were shared with the liver sample contributed to 69% (1720/2473) of the peaks identified in the E2-independent data set. These results indicated that in hepatocytes, ESR1 localization occurs through both ligand-dependent (lost and gained) and ligand-independent mechanisms.

**Figure 2:**
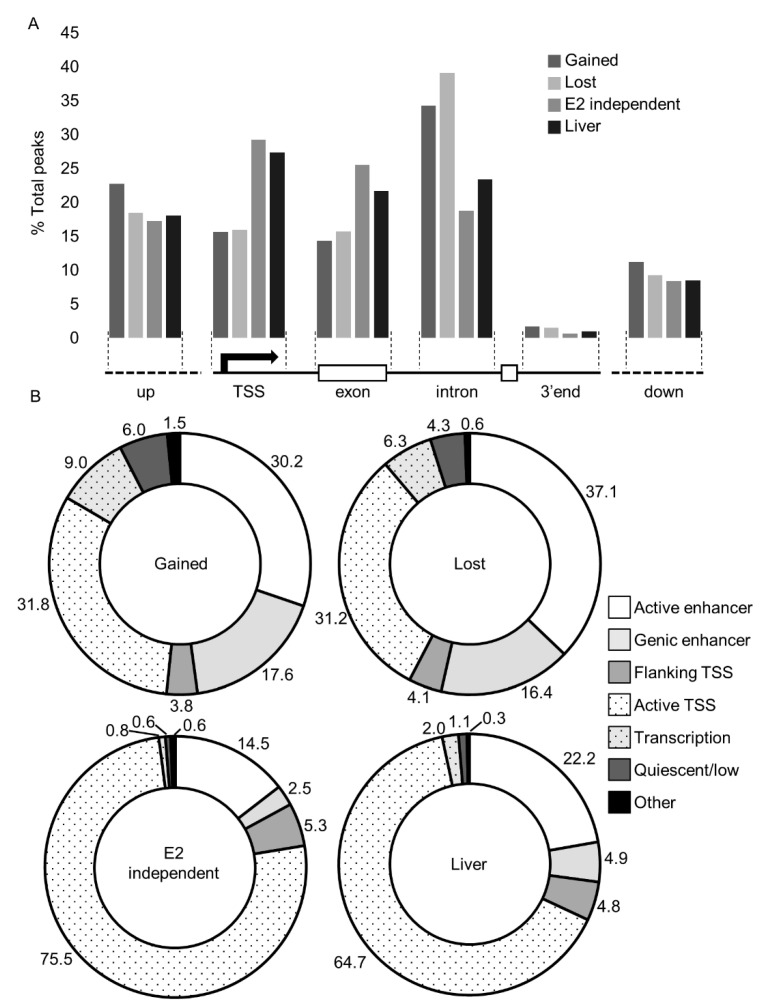
Genomic localization of ESR1. (**A**) Spatial localization determined by comparison of peaks to gene annotation in the UCSC genome browser. Values are percentages of total peaks in each region from each sample. Location is depicted in the cartoon below the graph, in the following order (left to right): upstream (−500 to −10 kb of TSS, up), proximal promoter (±2 kb of transcription start site, TSS), exonic, intronic, 3′-end (±500 bp from transcription end site), and downstream (+500 to +10 kb of TES, down). (**B**) Localization as determined by epigenetic histone marks. Values are percentages of total peaks. ChromHMM [23] was trained with data from liver samples and used to categorize the ESR1 peaks. TSS, transcription start site.

Next, we evaluated the genomic distribution of ESR1 in response to estrogen and in the liver tissues. We first utilized genome annotation and the UCSC Genome Browser [22] to spatially assign peaks relative to transcription start sites (TSS’s), exons, and introns (Figure 2A). In the lost and gained data sets, approximately 15% of ESR1 binding occurred within promoters (±500 bp). In the E2-independent and liver datasets, binding in promoter regions was roughly double (30%) the amount in the E2-responsive datasets. Comparison of ESR1 spatial localization between the datasets found that the E2-independent peaks show significantly different distribution compared to the lost (*p* = 0.019, χ^2^ test) and gained (*p* = 0.021, χ^2^ test) categories, but that E2-independent is not different from the liver sample. All four datasets had comparable enrichment upstream and downstream of genic regions. However, within gene transcribed regions, the E2-dependent datasets aligned primarily within introns (32% and 37% in gained and lost, respectively), whereas E2-independent binding and ESR1 binding in the liver was more evenly distributed across both exons and introns.

Studies in MCF-7 cells have illustrated that ESR1 binding primarily associates with enhancers [17]. To infer the genomic landscape of ESR1 binding, we utilized the ChromHMM algorithm [23] trained with ChIP-seq data on histone marks conducted in liver tissue and then compared our peak lists with the resulting ChromHMM model (Figure 2B). Ligand-dependent peaks were primarily associated with enhancers (47.8% and 53.5%, in gained and lost, respectively). In contrast, peaks associated with ligand-independent binding and those in liver tissues were more commonly located near TSSs (75.5% and 64.7%, respectively). These results largely agree with the spatial proximity-based approach (Figure 2A) and indicate distinct binding profiles for ligand-dependent and ligand-independent ESR1.

### 2.2. ESR1 Peaks Colocalize with a Variety of Trans-Factors

**Figure 3:**
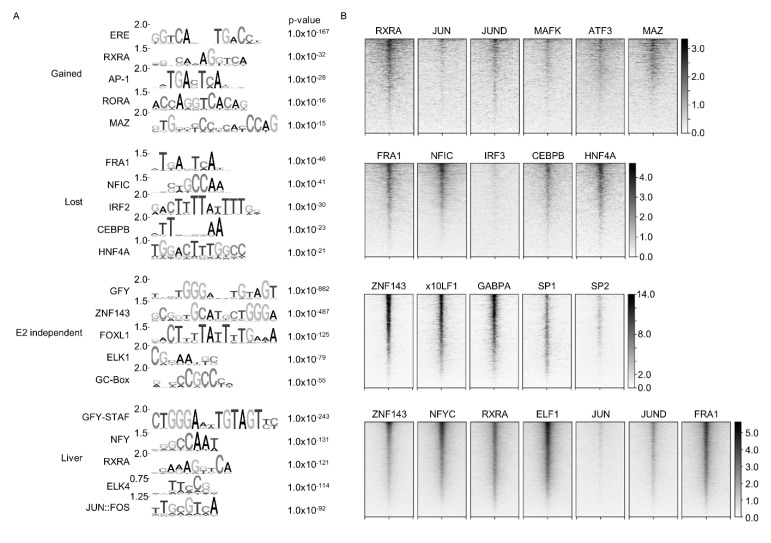
Motif enrichment and colocalization of transcription factors with ESR1 peaks. (**A**) The top five motifs in each sample are shown. The top bit value score is indicated to the left of each motif diagram. The p-value indicates enrichment of the motif compared to background. (**B**) Heatmaps showing ChIP-Seq read density centered on ESR1 peaks within a ±5 kb window. TF ChIP-Seq data were obtained from published datasets conducted in HepG2 cells. Darker intensity indicates higher enrichment, as indicated by the legends to the right of the plots.

Previous publications have shown that in addition to binding the canonical ERE, ESR1 is commonly associated with the motifs of other TFs. For example, ESR1 peaks in MCF-7 cells also contain motifs for forkhead box protein A1 (FOXA1) and activating enhancer binding protein 2 gamma (AP2γ) [16,17], indicating the involvement of other TFs in ESR1-DNA binding. Analysis of motifs enriched in the ligand-dependent, independent, and liver samples revealed enrichment of several different co-factor motifs (Figure 3A). Peaks that were lost after treatment contained motifs associated with a variety of TFs: fos-related antigen-1 (FRA1), nuclear factor I C (NFIC), CCAAT/enhancer-binding protein beta (CEBPB), interferon regulatory factor 2 (IRF2), and hepatocyte nuclear factor 4 alpha (HNF4A). Gained sites primarily contained the ERE, in agreement with the canonical ligand-induced localization of ESR1 [16,17]. Gained peaks also contained several less-enriched motifs including the retinoid X receptor alpha (RXRA), activator protein 1 (AP-1), RAR related orphan receptor A (RORA), and MYC associated zinc finger protein (MAZ). It is worth noting that both the RXRA and RORA motifs are ERE “half-sites”, which have been identified in previous ESR1 ChIP-Seq experiments [16,17]. In the ligand-independent peaks, the general factor Y (GFY) element and zinc finger protein 143 (ZNF143) were both highly enriched, followed by the forkhead box L1 (FOXL1), ETS-domain protein 1 (ELK1), and the GC-box. In the liver sample, the most enriched motif was the GFY-Staf motif, which at its core contains the GFY motif also found in the E2-independent peaks. The liver peaks also contained motifs recognized by the nuclear transcription factor Y (NFY), RXRA, ELK4, and JUN:FOS. Overall, these results suggest that ESR1 functions in the liver similarly to MCF-7 cells [17], binding the ERE in the presence of estrogen and also associating with other motifs, possibly indirectly via cofactors.

To determine whether the motifs enriched in the ESR1 peaks were bona fide binding sites in liver cells, published ChIP-Seq datasets with different TFs in HepG2 cells [24,25] were analyzed. Overall, we observed concomitant occurrence of these TFs with their corresponding motifs in the ESR1 peaks, supporting colocalization of these TFs with ESR1 (Figure 3B). In some cases, due to unavailability of ChIP-Seq data with a particular TF, similar TFs were chosen to act as surrogates for the enriched motifs. For instance, AP-1 is a complex of proteins that can potentially contain the JUN, FOS, MAF, and/or ATF proteins [26]. In the gained sample, JUN, JUND, MAFK, and ATF3 showed varying degrees of co-enrichment with the ESR1 peaks, possibly reflecting ESR1 preference for the different AP-1 complex constituents. In general, compared to the other ESR1 categories (lost, independent, and liver), the gained peaks showed more diffuse and less centralized TF colocalization, likely because ESR1 is directly binding to the ERE.

In the lost peaks, four TFs (FRA1, NFIC, CEBPB, and HNF4A) associated with the top five motifs all showed high enrichment at the ESR1 peak center (Figure 3B). IRF3 was chosen as a substitute for IRF2, as there was no HepG2 IRF2 dataset available and the IRF family of proteins all recognize a similar DNA motif [27]. However, there was little enrichment of IRF3 at the lost peaks, which may be due to IRF2 and IRF3 having different regulatory targets [28], thereby making IRF3 a poor alternative for visualizing IRF2 localization.

For the liver and ligand-independent samples, analysis of a factor binding the top GFY and GFY-Staf motifs in each sample was not feasible, as there is no definitive factor that binds this motif [29]. Still, ZNF143 showed high co-enrichment in the peak center of the ligand-independent peaks. No ELK1 dataset was available, so ELF1 and GABPA were chosen as substitutes due to their high motif similarity and SP1 and SP2 were chosen for the GC-Box as they are well-characterized GC-Box binding proteins [30]. ELF1, GABPA, and SP1 all showed high centralized colocalization with the ligand-independent ESR1 peaks.

The liver sample showed high centralized enrichment with NFYC, RXRA, ELF1 (ELK4), and JUND, JUN, and FRA1 (JUN::FOS). While conducting these colocalization analyses, it became more apparent how similar the collection of motifs in the liver was compared to those found in the three ESR1 binding conditions in hepatocytes. Like the gained peaks, the liver sample showed higher enrichment of JUND than of JUN and strong enrichment of RXRA. FRA1 selected as an ancillary JUN::FOS protein, showed strong central colocalization, and was also the top motif identified in the lost peaks. Finally, the liver sample also showed dense ELF1 and ZNF143 enrichment like the E2-independent sample. Although ZNF143 was not an identified motif in the liver sample per se, it was visualized due its partial motif overlap with GFY-Staf (the top identified motif in the liver). Overall, it appears that in the liver, ESR1 colocalizes with a variety of factors observed in both the ligand-dependent and independent datasets, reflecting a more varied localization possibly due to varying estrogen levels occurring in the combined liver sample.

It is noted that many of the co-enriched TFs show overlap with the various conditions despite not being in the top 5 motifs identified for that specific condition (Supplemental Figure S2). Many of these TF motifs are highly similar and/or are at a higher p-value in other ESR1 binding conditions. Overall, the results support indirect ESR1 binding (i.e., not through direct ERE interaction) at hepatic/liver chromatin through interaction with multiple proteins.

**Figure 4:**
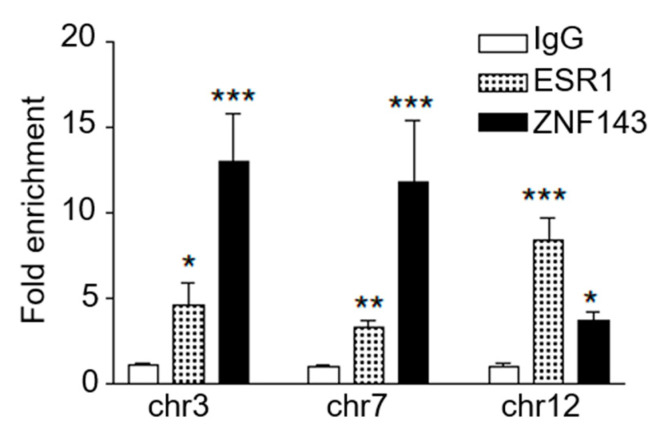
ChIP-qPCR confirmed enrichment of ESR1 and ZNF143 at three genomic sites identified in the E2-Independent sample. Data is shown as fold enrichment over the IgG control. Asterisks indicate statistically significant difference between ESR1/ZNF143 and the IgG control (ANOVA with Bonferroni post-test, * *p* < 0.05; ** *p* < 0.01; *** *p* < 0.001). See Supplemental Table S5 for information about the three chromatin targets.

To validate ESR1 and ZNF143 localization in liver cells, we conducted ChIP-qPCR and tested for both ESR1 and ZNF143 enrichment at three genomic locations in the E2- Independent dataset containing ZNF143 motifs (Figure 4). We confirmed ESR1 enrichment (3- to 8-fold) at these sites. Moreover, as expected, we also observed strong ZNF143 enrichment (4- to 13-fold) at each site. These results support colocalization of ZNF143 at ESR1 ChIP-Seq peaks in the absence of E2. Whether ESR1 binds to ZNF143 motifs directly or indirectly through interaction with ZNF143 or other factors requires further investigation.

### 2.3. Comparison to Other Cell Types

**Figure 5:**
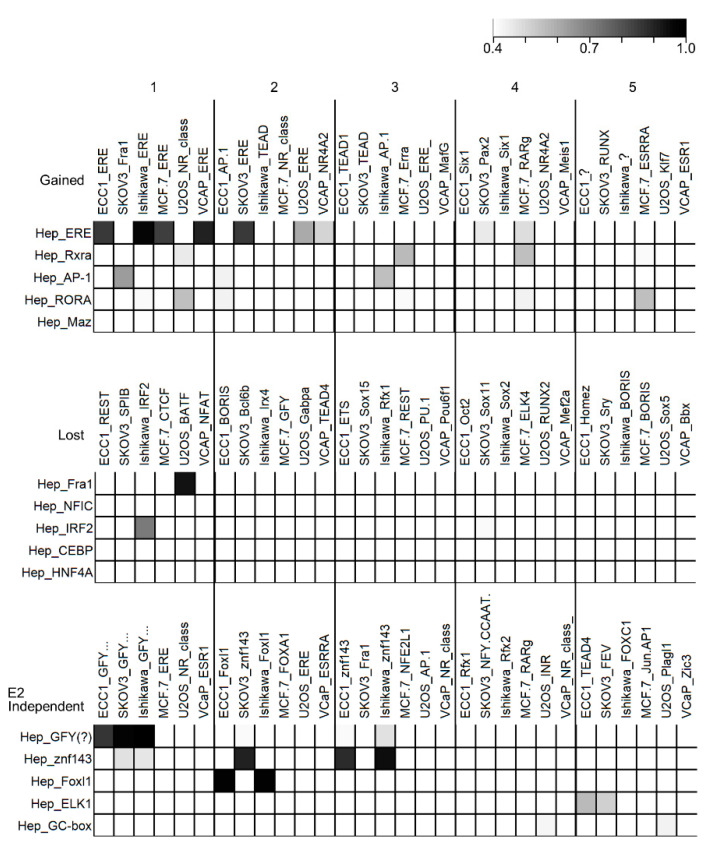
*Comparison of enriched motifs identified for ligand-responsive and ligand-independent ESR1 peaks in various cell lines.* Rows and columns are named with the cell type followed by the motif name (unknown motifs are labeled as “?”). Comparison matrices are separated by the ESR1 binding category (Gained, Lost, and E2 independent). Within each matrix, motifs from each cell line are compared to the top five motifs identified in the liver cells (row names, left). Each group is further divided into descending subgroups (left to right), with subgroup 1 being the #1 most enriched motif for the other six cell lines (column names) and subgroup five is the fifth most enriched motif (indicated by the numbers at the top of the figure). Darker cells indicate more similar motifs, ranging from identical (1) to not similar (0.4 or less), shown at the top right of the figure. Hep, hepatocytes.

The results from the motif enrichment and TF co-localization analyses suggested that ESR1 association with some factors in the liver is determined by the ligand (e.g., FRA1 only exists prior to treatment) while others exist regardless of E2 treatment (e.g., ZNF143). To determine whether these dynamic and static TF-TF interactions were unique to the liver, we analyzed six additional cell line ESR1 ChIP-Seq datasets: endometrial cancer (ECC1) [24], endometrial adenocarcinoma (Ishikawa) [31], prostate cancer (VCaP) [32], osteosarcoma (U2OS) [33], ovarian cancer (SKOV3) [34], and breast cancer (MCF-7) [35]. ESR1 peaks from these samples were similarly separated into gained, lost, and E2-independent categories (Supplemental Table S2). Motif enrichment analyses were conducted on each group and the top five motifs from each category were compared to the motifs we observed in hepatocytes (Figure 5). To compare motifs, we used the universal motif package [36] which provides a p-value reflecting the similarity of two or more motif matrices. In the gained peaks, as expected, the predominately enriched motif in all cell types was the ERE. When comparing the lost peaks, there was little motif similarity which suggests that prior to ligand binding, ligand-dependent ESR1 chromatin occupancy is determined by different cofactors in different cell lines, which may contribute to cell-specific gene regulation by ESR1. Interestingly, in the ligand-independent peaks, we found that the GFY motif was the most enriched motif in ECC1, SKOV3, and Ishikawa cell lines, identical to what we observed in hepatocytes (Figure 5). Furthermore, the independent peaks in the three cell lines were also enriched for the ZNF143 motif, and the ECC1 and Ishikawa cell lines also contained the Foxl1 motif. These results support a common underlying mechanism promoting ligand-independent localization of ESR1 to chromatin.

### 2.4. Pathways Associated with ESR1 Genomic Localization

To determine the pathways regulated by ESR1 in the liver, gene ontology (GO) analysis of the datasets was conducted (Table 1). GO-terms in the estrogen-dependent peak datasets were enriched in liver-related processes: with gained pathways including response to insulin, alcohol metabolism, and lipoprotein activity while lost pathways were associated with bile acid metabolism and glucose transport. In the ligand-independent dataset, pathways were related to ncRNA metabolism and protein synthesis. Pathways in the liver sample were most like those found in the ligand-independent group and included mRNA turnover and protein synthesis. These results suggest that ESR1 may play two broad roles in the liver: one where dynamic localization in response to E2 functions to regulate key liver-specific processes and another ligand-independent role that controls more general cellular activities.

### 2.5. ESR1 Localizes Near Genes Involved in Drug Metabolism

Previously, we demonstrated that ESR1 functions as a key regulator of several cytochrome P450 genes (CYPs), namely CYP3A4, CYP3A5, CYP3A7, CYP3A43, CYP2C9, CYP2C19, and CYP2A6 [18]. In support of this, we found ESR1 peaks within 50 kb of the TSS for all of them, except for CYP2C19 (Supplemental Table S3). To further investigate the role of ESR1 in drug metabolism, we generated a list of 170 pharmacogenes, including drug metabolizing enzymes, membrane transporters, liver-enriched TFs, and enzymes related to estrogen metabolism. We searched for ESR1 peaks within 50 kb of the TSS of these genes and identified ESR1 binding sites located near genes for 39/44 phase I and 37/55 phase II drug metabolism enzymes, 8/11 membrane transporters, 13/14 estrogen metabolism-related enzymes, and 40/46 TFs with known regulatory roles in the liver (Supplemental Table S3). These results further expand the role of ESR1 in regulating drug metabolism and liver gene expression.

## 3. Discussion

Our results found extensive ligand-free and ligand-mediated ESR1 binding in liver cells. By characterizing the localization of ESR1 in response to estrogen treatment, we further categorized ligand-dependent ESR1 binding sites as either gained or lost after treatment. These estrogen-responsive sites primarily occurred within enhancers, with gained locations indicating direct interaction of ESR1 with the ERE and lost sites instead suggesting indirect cofactor recruitment of ESR1 to chromatin. We also identified ESR1 binding sites that were maintained both before and after treatment, which we label here as ligand-independent binding sites. These ligand-independent sites suggest stable cofactor recruitment of ESR1 at gene promoters regardless of ligand availability. Both ligand-dependent and ligand-independent ESR1 binding sites were identified near genes involved in drug metabolism and transport, estrogen biosynthesis, and key TFs in the liver, consistent with our previous results showing ligand-free ESR1 as a master regulator for the expression of many CYPs [18], supporting the notion that ESR1 may play important roles in drug metabolism in the presence and absence of estrogen.

### 3.1. ERE, Ligand-Free, or Ligand-Independent ESR1 Chromatin Binding

Genome-wide ligand-free ESR1 localization in MCF-7 cells has been well described in two publications [16,17]. Caizzi et al. reported enrichment of both full and half-length ERE’s in ligand-free peaks, along with several other cofactors, such as FOXA1 and AP2γ. Bojcsuk and Bálint showed that a large subset of ESR1 sites (>65% of all identified ESR1 peaks) are bound by ligand-free ESR1, lack the ERE, and are instead enriched with co-factor motifs. In hepatocytes, we also found that ESR1 primarily associates with chromatin in a ligand-free fashion, with 77% of the peaks described in the differential binding comparison occurring in the lost and ligand-independent categories (Figure 1B). However, we should draw attention to the greater number of peaks identified in the untreated versus treated hepatocytes (Figure 1A), which may reflect genuine differential ESR1 chromatin enrichment in the two conditions or could potentially be driven by differences in ChIP-Seq sample processing. In support of differential ESR1 enrichment in the two samples, Caizzi et al. also observed 30% fewer ESR1 peaks after E2 treatment in MCF-7 cells [16]. Also, replicate experiments would likely have improved our ability to determine the cause of the different overall ESR1 peak numbers, but primary hepatocytes are a limited resource, and we chose to pool the samples to limit the effect of individual donor biases. Analysis of the ligand-free peaks showed that the ligand-independent peaks did not contain either a full or half-length ERE, lost sites only showed low enrichment of the half-ERE (data not shown), and the full-length ERE was only detected after treatment (Figure 3). Finally, the liver sample more closely resembled the ligand-independent peaks in localization (Figure 2), motif-enrichment (Figure 3), and functional annotation (Table 1). Taken together, our results indicate that indirect localization of ESR1 to the liver genome is fundamental for regulation of gene expression, consistent with the results reported by Bojcsuk and Bálint [17].

### 3.2. ESR1 Mediated Gene Regulation in the Liver Occurs through Cofactors and Direct-DNA Binding

ESR1 binding post-treatment to the ERE has well-characterized roles in gene activation or repression [37]. Also, indirect binding of ESR1 to DNA is integral for its regulation of gene expression, with pioneer factors (e.g., FOXA1, AP2γ) playing a key role in ESR1 binding to chromatin [38]. Similar to its dual role in regulating genes when directly bound to the ERE, ESR1-containing complexes have been shown to differentially regulate genes lacking an ERE. For instance, SP-1 can bridge ESR1 to DNA in the absence of estrogen, and estrogen treatment leads to co-activator recruitment and gene activation [39,40,41]. Conversely, a complex containing ligand-free ESR1 at the promoter of the tumor necrosis factor alpha (*TNFα*) which activates transcription and treatment instead leads to its repression [14]. Because ESR1 regulation is context specific, further studies to dissect the regulatory roles of ESR1 in the liver are warranted.

Regardless of the specific effects on transcription, our results showing co-enrichment of several TFs with ESR1 indicate indirect binding of ESR1 throughout the liver genome. In further support of this, interaction with ESR1 has been shown for many of the co-enriched TFs identified in this study: RXRA, HNF4A, CEBPB, SP1 and SP2, subunits of the AP-1 complex (JUN, JUND, and MAZ), GABPA, and the NFY complex [41,42,43,44,45,46,47,48,49]. Some of these (RXRA, HNF4A, and CEBPB) are known to regulate the expression of pharmacogenes, consistent with our results showing ESR1 localization near their promoters (Supplemental Table S3).

Of note, ligand-independent ESR1 binding sites were highly enriched for the ZNF143 motif in liver and other reproductive tissues and our ChIP-qPCR experiments validated significant colocalization of ZNF143 at three E2-independent sites. ZNF143 has known roles in chromatin looping [50] and it appears to play an essential role in ESR1-mediated gene regulation in MCF-7 cells [51], suggesting that ZNF143 and ESR1 may establish three-dimensional chromatin landscapes connecting distal enhancers and promoters to regulate gene expression. Furthermore, ZNF143 was among several other ligand-independent cofactors enriched in cancer cell lines derived from ovarian and uterus tissues (Figure 5). Interestingly, in an ESR1 ChIP-Seq experiment conducted in mouse liver tissue, the Staf motif (a component of the ZNF143 motif) was enriched in placebo-treated but not E2-treated mouse livers [52]. However, our analyses of the mouse liver data did not identify the ZNF143 or GFY-Staf motifs in the E2-independent dataset, and instead identified low enrichment of ZNF143 in the mouse ESR1 gained category (data not shown). Whether colocalization of ZNF143 and ESR1 is conserved in mammals will require further experimentation. Thus, our results suggest general roles of ESR1 in establishing or stabilizing enhancer-promoter interactions, potentially playing an important role in regulating gene expression. Experiments describing the interaction between ESR1 and ZNF143 and their potential regulation of gene expression through chromatin looping are an intriguing future direction.

### 3.3. Implications for ESR1 Regulation in the Liver

ESR1 is known to be a key transcriptional regulator in the liver [6]. In mouse livers, ESR1 was found to bind fewer sites compared to what had previously been observed in human cells [52]. Our experiments found that in human livers, the number of ESR1 binding sites has greater similarity to that observed in MCF-7 cells [16] and is comparable to other cell lines analyzed in this study (Supplemental Table S2). These binding sites were associated with known liver pathways, such as lipid and insulin metabolism, and more general pathways, such as RNA and protein metabolism (Table 1). In contrast, in mouse livers, ESR1 peaks were predominately associated with liver-specific pathways [52]. Overall, our results suggest an expanded role for ESR1 regulation in human livers and may help explain some of the observed differences in gene expression between humans and mice. For instance, there are several species-specific sex-driven differences in expression of the CYP enzymes [12,13,53,54] and studies have illustrated that human and mouse liver gene expression profiles are highly different, more so than when compared to other tissues in the same species [55,56].

We also identified peaks near important pharmacogenes, genes with roles in estrogen metabolism, and key liver TFs (Supplemental Table S3). Therefore, genetic, epigenetic, and non-genetic (e.g., tamoxifen or fulvestrant, ESR1 targeting drugs) factors that affect ESR1 function and/or expression may contribute significantly to the liver transcriptome, drug metabolism, and liver diseases. Interestingly, ESR1 splice isoforms lacking the N- terminal domain (ERα46) are predominant in the liver [57] and several ESR1 isoforms containing truncated C-terminal domains with known altered transactivation and DNA- binding profiles [58,59] are also found in the liver [57]. Moreover, expression of these ESR1 isoforms in the liver is highly variable between individuals [57]. In this study, liver samples were pooled from three males and three females to limit the effect of individual samples on ESR1 localization. Future experiments documenting differences between ESR1 localization in males and females may further our knowledge of the role of ESR1 in driving sexual-dimorphism in the liver. Therefore, the potential alteration of ESR1’s regulatory function due to splicing variants may contribute to the well-documented differences in drug response and disease susceptibility between individuals.

## 4. Conclusions

Our ESR1 ChIP-Seq results show discrete genomic binding sites for ligand-free and ligand-bound ESR1 that contain distinct binding motifs and biological pathways in human liver cells, supporting both estrogen-dependent and independent regulatory roles of ESR1. Moreover, we found frequent genomic localization of ESR1 near many pharmacogenes, suggesting the potential broad influence of ESR1 on drug metabolism and drug therapy.

## 5. Methods

### 5.1. Liver Tissues and Hepatocyte Samples

The demographic information for the six liver tissues (three males and three female, age 44 to 73) is provided in Supplemental Table S1. Liver tissues from the six donors were pooled and used for ChIP-Seq. Cryopreserved human primary hepatocytes from two donors (both European American, one male and one female, age 32 and 62, respectively) were obtained from Lonza (Basel, Switzerland).

### 5.2. Cell Culture and Treatment

Cryopreserved human hepatocytes were recovered and cultured according to the protocol provided by Lonza. Briefly, cells were thawed and washed once with thawing medium (MCHT50, Lonza, Basel, Switzerland). Then the cells were resuspended in plating medium (MP100, Lonza, Basel, Switzerland), counted, and plated in 6-well collagen coated plates. The cells were cultured in a 37 °C/5% CO2 incubator. Four hours later, plating medium was replaced with maintenance medium, containing William’s E media supplemented with penicillin/streptomycin/fungizone (100 U/100 µg/0.25 µg per mL), 100 nM dexamethasone, 2 mM L-glutamine, 15 mM HEPES, and ITS (0.55 mg/mL human transferrin, 1 mg/mL bovine insulin and 0.5 µg/mL sodium selenite, from Sigma Aldrich, St. Louis, MO, USA). Eighteen hours after in culture, cells were treated with DMSO or 17-beta-estradiol (E2, 1 µM) for 8 h, this relatively high dosage was chosen because a previous report demonstrated that E2 is rapidly metabolized in hepatocytes and that they require a higher dosage than other cell lines [20]. The cells were then cross-linked for 15 min with 1% formaldehyde. Cells from the two donors were pooled and used for ChIP-Seq.

### 5.3. Chromatin Immunoprecipitation Followed by High Throughput Sequencing (ChIP-Seq)

ChIP-Seq experiments on fixed hepatocytes and frozen liver tissues were performed using a commercial service (Active Motif, Carlsbad, CA, USA), which includes preparation of chromatin from fixed cells and liver tissues, sonication, ChIP with an anti-ESR1 antibody, ChIP-Seq library preparation, next-generation sequencing, and delivery of fastq sequencing data. The anti-ESR1 antibody (sc-543) was obtained from Santa Cruz biotechnology (Santa Cruz Biotech, Dallas, TX, USA).

### 5.4. Data Analysis

All datasets were processed using the same pipeline. Raw fastq files were analyzed with FastQC (v0.11.7) [60] and Trimmomatic (v0.39) [61] was used to remove adaptors, bps with a quality score less than 20, and sequences less than 20 bp in length [61]. Resulting fastq files were aligned to the hg38 genome [62] using Bowtie2 (v2.3.5.1) [63] with the local alignment mode, which allows the reads to be trimmed from both ends to maximize the alignment score. Aligned files were then processed using the factor mode of Homer v4.10 [64] to identify peaks, conduct motif enrichment analyses, and to produce bigwig files. Untreated and E2-treated peaks were generated by comparison to input genomic DNA derived from hepatocytes (Hep input). The liver peaks were determined by comparison to input genomic DNA from liver (Liver input). Shared peaks were defined as any peaks that had an overlap of at least one bp.

Homer v4.10 [64] was also used to determine differential ESR1 peaks. Differential peaks are those that are at least four-fold different between samples (default Homer settings). Gained peaks were determined by calling peaks on the E2-treated sample with the untreated sample as background. Any peaks that were not found in the E2-treated peak analysis were removed (i.e., not found in E2-treated vs. Hep input). Lost peaks were determined by calling peaks on the untreated sample with the E2-treated sample as background and any peaks that were not found in the untreated peak analysis were removed. In both cases, removal of these peaks was to ensure no false background peaks were included in the analysis. Peaks that were found in both untreated and E2-treated samples (within a range of four-fold) were considered E2-independent.

ChromHMM (v1.19) [23] and UCSC genome annotations (GRCh38.p13) [65] were used for annotating the ChIP-Seq peaks. ChromHMM was trained with data from human liver samples (H3K36me3, H3K27ac, H3K4me1, H3K4me3, H3K27me3, H3K9me3) obtained from the human reference epigenome project [66]. The R packages universalmotif (v1.4.9) [36] and ggseqlogo (v0.1) [67] were used to conduct motif comparisons and generate matrix images. ChIP-Seq density plots were generated from the bigwig files using deepTools2 (v3.1.1) [68]. GREAT (v4.0.4) [69] was used to generate GO terms for the top 5000 peaks in each dataset and REVIGO (http://revigo.irb.hr/) [70] was used to condense redundant GO terms. Datasets used in this study are listed in Supplemental Table S4.

### 5.5. Chromatin Immunoprecipitation Followed by qPCR (ChIP-qPCR)

ChIP was performed in primary human hepatocytes using the ChIP-IT^®^ Express Enzymatic kit (Active Motif, Carlsbad, CA, USA) with an anti-ESR1 (sc-8002x, Santa Cruz Biotech, Dallas, TX, USA) or anti-ZNF143 (sc-100983, Santa Cruz Biotech, Dallas, TX, USA) antibody as described [71]. ESR1 or ZNF143 enriched fragments were measured with real-time PCR using SYBR Green and specific primers (chr3-FP&RP: CTAGCTGCGCGTAGAGCAC, CATAGTTCAGCCAGCGCGTC; chr7-FP&RP: CCTGGAGGGAGACATAGCG, CCGGAAGCGGCTAAGACAC; chr12-FP&RP: GGACTTGTAGTCTCCCACGC, GGACGGTAGTCACACGACAG) and fold enrichment was calculated relative to a negative control prepared with nonspecific IgG antibody. Genomic information for the ChIP-qPCR targets is described in Supplemental Table S5).

## Figures and Tables

**Table 1:** GREAT analysis of ESR1 ChIP-Seq peaks, top five terms are shown.

Sample	Description	BinomFdrQ	GO Term
Gained	cellular response to insulin stimulus	4.47 × 10^−6^	GO:0032869
	alcohol metabolic process	7.15 × 10^−6^	GO:0006066
	cell junction assembly	6.21 × 10^−4^	GO:0034329
	regulation of lipoprotein lipase activity	3.14 × 10^-3^	GO:0051004
	multicellular organism growth	1.32 × 10^−2^	GO:0035264
Lost	regulation of intrinsic apoptotic signaling pathway	4.36 × 10^−5^	GO:2001242
	bile acid metabolic process	7.14 × 10^−5^	GO:0008206
	glucose transport	2.62 × 10^−4^	GO:0015758
	embryonic retina morphogenesis in camera-type eye	7.28 × 10^−3^	GO:0060059
	reactive nitrogen species metabolic process	1.59 × 10^−2^	GO:2001057
Independent	ncRNA metabolic process	1.25 × 10^−11^	GO:0034660
	translation	3.78 × 10^−9^	GO:0006412
	protein targeting to ER	1.57 × 10^−8^	GO:0045047
	response to endoplasmic reticulum stress	1.40 × 10^−6^	GO:0034976
	ciliary basal body docking	2.62 × 10^−3^	GO:0097711
Liver	nuclear-transcribed mRNA catabolic process	8.56 × 10^−17^	GO:0000956
	ER-nucleus signaling pathway	5.88 × 10^−6^	GO:0006984
	mitochondrial transmembrane transport	1.39 × 10^−5^	GO:1990542
	apoptotic mitochondrial changes	4.65 × 10^−5^	GO:0008637
	’de novo’ protein folding	1.37 × 10^−3^	GO:0006458

## Data Availability

The data has been deposited in the gene expression omnibus under GSE158856.

## Supplementary Materials

The following are available online at https://www.mdpi.com/1422-0067/22/3/1461/s1. Table S1. Sample demographic information. Table S2. ESR1 ChIP-Seq peaks identified in all experiments. Table S3. ESR1 ChIP-Seq peaks located within 50 kb of selected liver genes. Table S4. Datasets used in this study. Table S5. ChIP-qPCR peak information. Figure S1. ChIP-Seq peak examples. The three different panels are selected to show ChIP-Seq peaks used to categorize ESR1 binding into lost, gained, and E2-independent datasets. Asterisks indicate peaks that are E2-independent and are found in both untreated and E2-treated samples. Solid-filled circles indicate peaks that are either gained (located in E2-treated only) or lost (located in untreated only). Figure S2. Transcription factor colocalization across all ESR1 binding categories. Heatmaps illustrating ChIP-Seq read density centered on ESR1 peaks within a ±5 kb window. The figure is organized into four rows separated into four blocks. Each row shows TF density around a specific ESR1 binding category (gained, lost, E2- independent, and liver). Each vertical line separates TFs in blocks that were enriched in each specific ESR1 category. Plots highlighted with a dark border were identified in both experiments (e.g., RXRA was identified in both gained and liver). TF ChIP-Seq data were obtained from published datasets conducted in HepG2 cells. Darker intensity indicates higher enrichment, as indicated by the legends to the right of the plots.

## Abbreviations

AP-1	Activator protein 1
AP2γ	Activating enhancer binding protein 2 gamma
ATF	Activating transcription factor
ATF3	Activating transcription factor 3
CEBPB	CCAAT/enhancer-binding protein beta
ChIP-Seq	Chromatin immunoprecipitation followed by next generation sequencing
CYP	Cytochrome P450
DMSO	Dimethyl sulfoxide
DNA	Deoxyribonucleic acid
E2	17-beta-estradiol
ELF1	E74 like ETS transcription factor 1
ELK1	ETS-domain protein 1
ELK4	ETS-domain protein 4
ERE	Estrogen response element
ESR1	Estrogen receptor alpha
FOS	Fos proto oncogene
FOXA1	Forkhead box protein A1
FOXL1	forkhead box L1
FRA1	fos-related antigen 1
GABPA	GA Binding Protein Transcription Factor Subunit Alpha
GFY	General factor Y
GO	Gene ontology
HNF4A	Hepatocyte nuclear factor 4
IgG	Immunoglobulin G
IRF2	Interferon regulatory factor 2
IRF3	Interferon regulatory factor 3
JUN	JUN protooncogene
JUND	JUN D protooncogene
MAF	MAF BZIP transcription factor
MAFK	MAF BZIP transcription factor K
MAZ	MYC associated zync finger protein
NFIC	Nuclear factor I C
NFY	Nuclear transcription factor Y
RNA	Ribonucleic acid
RORA	RAR related orphan receptor A
RXRA	Retinoid X receptor alpha
SP-1	Specificity factor 1
SP-1	Specificity factor 2
TES	Transcription end site
TF	Transcription factor
TNFα	Tumor necrosis factor alpha
TSS	Transcription start site
ZNF143	Zinc finger protein 143
