# Supplementary material for: ESR1 ChIP-Seq Identifies Distinct Ligand-Free ESR1 Genomic Binding Sites in Human Hepatocytes and Liver Tissue
Source: Int J Mol Sci. 2021 Feb 2;22(3):1461. doi: 10.3390/ijms22031461 (PMC7867289; doi:10.3390/ijms22031461)
Supplement: Supplementary file 1 [file ijms-22-01461-s001.zip › Supplemental_tables.docx]

Supplemental Table 1. Sample demographic information.

| Sample ID | Type | Age | Race | Gender |
| --- | --- | --- | --- | --- |
| L111 | Liver Tissue | 58 | Caucasian | Female |
| L115 | Liver Tissue | 44 | Caucasian | Male |
| L117 | Liver Tissue | 54 | Caucasian | Male |
| L119 | Liver Tissue | 67 | Caucasian | Male |
| L122 | Liver Tissue | 65 | Caucasian | Female |
| L125 | Liver Tissue | 77 | Caucasian | Female |
| Hep4111 | Primary Hepatocyte | 32 | Caucasian | Male |
| Hep4232 | Primary Hepatocyte | 62 | Caucasian | Female |

Supplemental Table 2. ESR1 ChIP-Seq peaks identified in all experiments.

| Tissue/Cell Type | E2 Dosage | Treatment Length | Sample Name | Peak Number |  |
| --- | --- | --- | --- | --- | --- |
| Liver | N/A | N/A | Liver | 14664 |  |
| Hepatocytes | 1 µM | 8 hr | E2 treated | 5587 |  |
|  |  |  | Untreated | 9510 |  |
|  |  |  | E2 Independent | 2473 |  |
|  |  |  | Gained | 1551 |  |
|  |  |  | Lost | 2825 |  |
| U2OS | 10 nM | 45 min | E2 Independent | 2556 |  |
|  |  |  | Gained | 58127 |  |
|  |  |  | Lost | 592 |  |
| Ishikawa | 10 nM | 1 hr | E2 Independent | 2253 |  |
|  |  |  | Gained | 8731 |  |
|  |  |  | Lost | 184 |  |
| VCaP | 10 nM | 14 hr | E2 Independent | 734 |  |
|  |  |  | Gained | 6869 |  |
|  |  |  | Lost | 280487 |  |
| ECC1 | 10 nM | 1 hr | E2 Independent | 5780 |  |
|  |  |  | Gained | 19225 |  |
|  |  |  | Lost | 578 |  |
| SKOV3 | 10 nM | 24 hr | E2 Independent | 3765 |  |
|  |  |  | Gained | 11348 |  |
|  |  |  | Lost | 99 |  |
| MCF-7 | 100 nM | 45 min | E2 Independent | 20804 |  |
|  |  |  | Gained | 1821 |  |
|  |  |  | Lost | 6634 |  |

Supplemental Table 3. ESR1 ChIP-Seq peaks located within 50 kb of selected liver genes.

| Gene category | Gained | Lost | Independent | Liver |
| --- | --- | --- | --- | --- |
| Drug Metabolizing CYPs | CYP1A1 | CYP2C8 |  | CYP1A1 |
|  | CYP1A2 | CYP2C9 |  | CYP1A2 |
|  | CYP1B1 | CYP3A4 |  | CYP1B1 |
|  | CYP2B6 | CYP3A43 |  | CYP2A6 |
|  | CYP2C18 |  |  | CYP2B6 |
|  |  |  |  | CYP2C18 |
|  |  |  |  | CYP2D6 |
|  |  |  |  | CYP2E1 |
|  |  |  |  | CYP3A5 |
|  |  |  |  | CYP3A7 |
| Other CYPs | CYP17A1 | CYP27B1 | CYP17A1 | CYP11A1 |
|  | CYP27B1 | CYP4F2 | CYP2R1 | CYP17A1 |
|  | CYP7B1 | CYP7A1 |  | CYP21A2 |
|  | CYP8B1 |  |  | CYP24A1 |
|  |  |  |  | CYP27A1 |
|  |  |  |  | CYP27B1 |
|  |  |  |  | CYP2J2 |
|  |  |  |  | CYP2R1 |
|  |  |  |  | CYP4B1 |
|  |  |  |  | CYP4F2 |
|  |  |  |  | CYP7A1 |
|  |  |  |  | CYP7B1 |
|  |  |  |  | CYP8B1 |
| Other phase I enzymes | EPHX1 | ALDH1A1 | FMO4 | ALDH1A1 |
|  |  | EPHX1 | NQO2 | ALDH2 |
|  |  | FMO4 |  | CES1 |
|  |  |  |  | CES2 |
|  |  |  |  | EPHX1 |
|  |  |  |  | EPHX2 |
|  |  |  |  | FMO2 |
|  |  |  |  | FMO4 |
|  |  |  |  | FMO5 |
|  |  |  |  | NQO1 |
|  |  |  |  | NQO2 |
| Phase II enzymes: UGTs |  | UGT1A10 |  | UGT1A1 |
|  |  | UGT1A3 |  | UGT1A10 |
|  |  | UGT1A4 |  | UGT1A3 |
|  |  | UGT1A5 |  | UGT1A4 |
|  |  | UGT1A6 |  | UGT1A5 |
|  |  | UGT1A7 |  | UGT1A6 |
|  |  | UGT1A9 |  | UGT1A7 |
|  |  | UGT2B11 |  | UGT1A9 |
|  |  | UGT2B4 |  | UGT3A1 |
| Phase II enzymes: Sulfotransferases | SULT1B1 | SULT1E1 | SULT1A2 | SULT1A1 |
|  |  |  | SULT2B1 | SULT1A2 |
|  |  |  |  | SULT1B1 |
|  |  |  |  | SULT2A1 |
|  |  |  |  | SULT2B1 |
|  |  |  |  | SULT4A1 |
|  |  |  |  | SULT6B1 |
| Phase II enzymes: GSTs | GSTP1 | GSTA1 | GSTA4 | GSTA2 |
|  |  | GSTA2 | GSTO1 | GSTA3 |
|  |  | GSTO1 | GSTO2 | GSTA4 |
|  |  | GSTO2 | GSTP1 | GSTK1 |

Supplemental Table 3. Continued.

| Gene category | Gained | Lost | Independent | Liver |
| --- | --- | --- | --- | --- |
| Phase II enzymes: GSTs continued |  | GSTP1 | GSTZ1 | GSTM1 |
|  |  | MGST1 | MGST2 | GSTM2 |
|  |  |  |  | GSTM3 |
|  |  |  |  | GSTM4 |
|  |  |  |  | GSTM5 |
|  |  |  |  | GSTO1 |
|  |  |  |  | GSTO2 |
|  |  |  |  | GSTP1 |
|  |  |  |  | GSTZ1 |
|  |  |  |  | MGST1 |
|  |  |  |  | MGST2 |
|  |  |  |  | MGST3 |
| Transporters | SLC51A |  | ABCB1 | ABCB1 |
|  |  |  | ABCB11 | ABCB11 |
|  |  |  | SLC51B | ABCC3 |
|  |  |  | SLCO4C1 | ABCG5 |
|  |  |  |  | ABCG8 |
|  |  |  |  | SLC10A1 |
|  |  |  |  | SLC51A |
|  |  |  |  | SLC51B |
|  |  |  |  | SLCO4C1 |
| Estrogen metabolism-related | HSD3B1 | POR | HSD17B8 | HSD17B1 |
|  | POR | AKR1C3 | SCAP | HSD17B2 |
|  | SREBF2 |  |  | HSD17B4 |
|  |  |  |  | HSD17B7 |
|  |  |  |  | HSD17B8 |
|  |  |  |  | HSD3B1 |
|  |  |  |  | HSD3B2 |
|  |  |  |  | POR |
|  |  |  |  | SREBF1 |
|  |  |  |  | SREBF2 |
|  |  |  |  | SCAP |
|  |  |  |  | STAR |
| Liver-enriched TFs | ESR1 | DBP | DBP | AHRR |
|  | NCOA1 | HNF4G | FOXA3 | AHRR |
|  | NR3C1 | NCOR1 | NCOA3 | ARNT |
|  | ONECUT1 | NFE2L2 | NR0B2 | CEBPA |
|  | POR | NR1H2 | NR1D2 | CEBPB |
|  | PPARA | NR1H4 | NR1H2 | CEBPD |
|  | RXRA | NR2F2 | NR1H3 | CEBPG |
|  | YY1 | PPARA | ONECUT1 | DBP |
|  |  | PPARD | PPARD | ESR1 |
|  |  | THRB | RXRB | FOXA1 |
|  |  |  | THRA | FOXA3 |
|  |  |  | UGT1A8 | HNF4A |
|  |  |  |  | HNF4G |
|  |  |  |  | NCOA1 |
|  |  |  |  | NCOA2 |
|  |  |  |  | NCOA3 |
|  |  |  |  | NCOR1 |
|  |  |  |  | NCOR2 |
|  |  |  |  | NFE2L2 |
|  |  |  |  | NR0B2 |
|  |  |  |  | NR1D2 |

Supplemental Table 3. Continued.

| Gene category | Gained | Lost | Independent | Liver |
| --- | --- | --- | --- | --- |
| Liver-enriched TFs continued |  |  |  | NR1D2 |
|  |  |  |  | NR1H2 |
|  |  |  |  | NR1H3 |
|  |  |  |  | NR1H4 |
|  |  |  |  | NR1I2 |
|  |  |  |  | NR1I3 |
|  |  |  |  | NR2F1 |
|  |  |  |  | NR3C1 |
|  |  |  |  | NR5A2 |
|  |  |  |  | ONECUT1 |
|  |  |  |  | PPARA |
|  |  |  |  | PPARD |
|  |  |  |  | PPARG |
|  |  |  |  | RXRB |
|  |  |  |  | THRA |
|  |  |  |  | THRB |
|  |  |  |  | USF1 |
|  |  |  |  | VDR |
|  |  |  |  | YY1 |

Supplemental Table 4. Datasets used in this study.

| ChIP target | Cell-type | Dataset series | Input | Vehicle | Treatment | DOI |
| --- | --- | --- | --- | --- | --- | --- |
| ESR1 | MCF-7 | GSE25021 | ERR011971 | ERR011978 | ERR011973 | 10.1101/gr.100479.109 |
|  | U2OS | GSE26110 | SRR088773 | SRR088770 | SRR088769 | 10.1016/j.mce.2017.03.005 |
|  | Ishikawa | GSE109891 | SRR6653440 | SRR6653434 | SRR6653432 | 10.1016/j.celrep.2018.02.076 |
|  | VCaP | GSE43985 | SRR658532 | SRR658528 | SRR658530 | 10.1038/ncomms6383 |
|  | SKOV3 | GSE116005 | SRR7367337 | SRR7367341 | SRR7367339 | 10.7150/thno.30814 |
|  |  |  | SRR7367338 | SRR7367342 | SRR7367340 |  |
|  | ECC1 | GSE32465 | SRR351753 | SRR351676 | SRR351674 | 10.1016/j.molcel.2013.08.037 |
|  |  |  | SRR351754 | SRR351677 | SRR351675 |  |
| FRA1 | HepG2 | GSE32465 | SRR351753 | SRR351729 |  | 10.1016/j.molcel.2013.08.037 |
|  |  |  | SRR351754 | SRR351730 |  |  |
| NFIC | HepG2 | GSE32465 | SRR577837 | SRR577692 |  | 10.1016/j.molcel.2013.08.037 |
|  |  |  | SRR577838 | SRR577693 |  |  |
| IRF3 | HepG2 | GSE31477 | SRR502419 | SRR502708 |  | 10.1038/nature11247 |
|  |  |  | SRR502420 | SRR502709 |  |  |
| CEBPB | HepG2 | GSE32465 | SRR351753 | SRR577766 |  | 10.1016/j.molcel.2013.08.037 |
|  |  |  | SRR351754 | SRR577767 |  |  |
| HNF4A | HepG2 | GSE32465 | SRR351753 | SRR351747 |  | 10.1016/j.molcel.2013.08.037 |
|  |  |  | SRR351754 | SRR351748 |  |  |
| ELF1 | HepG2 | GSE32465 | SRR351753 | SRR351663 |  | 10.1016/j.molcel.2013.08.037 |
|  |  |  | SRR351754 | SRR351664 |  |  |
| GABPA | HepG2 | GSE32465 | SRR351751 | SRR351519 |  | 10.1016/j.molcel.2013.08.037 |
|  |  |  | SRR351752 | SRR351520 |  |  |
| SP1 | HepG2 | GSE32465 | SRR351859 | SRR351831 |  | 10.1016/j.molcel.2013.08.037 |
|  |  |  | SRR351861 | SRR351832 |  |  |
| SP2 | HepG2 | GSE32465 | SRR577837 | SRR577784 |  | 10.1016/j.molcel.2013.08.037 |
|  |  |  | SRR577838 | SRR577785 |  |  |
| RXRA | HepG2 | GSE32465 | SRR351859 | SRR351731 |  | 10.1016/j.molcel.2013.08.037 |
|  |  |  | SRR351861 | SRR351732 |  |  |
| JUND | HepG2 | GSE32465 | SRR351859 | SRR351817 |  | 10.1016/j.molcel.2013.08.037 |
|  |  |  | SRR351861 | SRR351818 |  |  |
| JUN | HepG2 | GSE31477 | SRR502419 | SRR502111 |  | 10.1038/nature11247 |
|  |  |  | SRR502420 | SRR502112 |  |  |
| ATF3 | HepG2 | GSE32465 | SRR351753 | SRR351823 |  | 10.1016/j.molcel.2013.08.037 |
|  |  |  | SRR351754 | SRR351824 |  |  |
| MAFK | HepG2 | GSE31477 | SRR502419 | SRR501991 |  | 10.1038/nature11247 |
|  |  |  | SRR502420 | SRR501992 |  |  |
| MAZ | HepG2 | GSE31477 | SRR502419 | SRR502055 |  | 10.1038/nature11247 |
|  |  |  | SRR502420 | SRR502056 |  |  |
| NFYC | HepG2 | GSE104247 | SRR5331462 | SRR5338972 |  | 10.1038/nature11247 |
|  |  |  |  | SRR5338973 |  |  |
| ZNF143 | HepG2 | GSE104247 | SRR5111848 | SRR5111095 |  | 10.1038/nature11247 |
|  |  |  | SRR5111849 | SRR5111096 |  |  |
| H3K27me3 | Liver | GSE16256 |  | SRR1143971 |  | 10.1038/nature14248 |
| H3K36me3 | Liver | GSE16256 |  | SRR643642 |  | 10.1038/nature14248 |
| H3K27ac | Liver | GSE16256 |  | SRR1143969 |  | 10.1038/nature14248 |
|  |  |  |  | SRR1143970 |  |  |
| H3K9me3 | Liver | GSE16256 |  | SRR1143975 |  | 10.1038/nature14248 |
| H3K4me1 | Liver | GSE16256 |  | SRR643651 |  | 10.1038/nature14248 |
| H3K4me3 | Liver | GSE16256 |  | SRR1143973 |  | 10.1038/nature14248 |
|  |  |  |  | SRR1143974 |  |  |

Supplemental Table 5. ChIP-qPCR peak information.

| Chr | Start | End | Distance to TSS | Gene Name | ZNF143 Motif(s)* |
| --- | --- | --- | --- | --- | --- |
| chr12 | 6852331 | 6852651 | 364 | USP5 | 55(GCGATGCACCATGGGA,+) |
| chr7 | 63926212 | 63926532 | -72434 | ZNF722P | 102(TCCCAGCATGCAACGG,-) |
|  |  |  |  |  | 166(GCCAGGCATGCTGGGA,+) |
|  |  |  |  |  | 236(TCCCAGCATGCAAGGG,-) |
| chr3 | 50292362 | 50292682 | 73 | IFRD2 | 255(ACCCACAATGCCCTGC,-) |

*Shown as distance (sequence,strand), distance is relative to start of peak.
