# Supplementary figures and images for: ESR1 ChIP-Seq Identifies Distinct Ligand-Free ESR1 Genomic Binding Sites in Human Hepatocytes and Liver Tissue
Source: Int J Mol Sci. 2021 Feb 2;22(3):1461. doi: 10.3390/ijms22031461 (PMC7867289; doi:10.3390/ijms22031461)

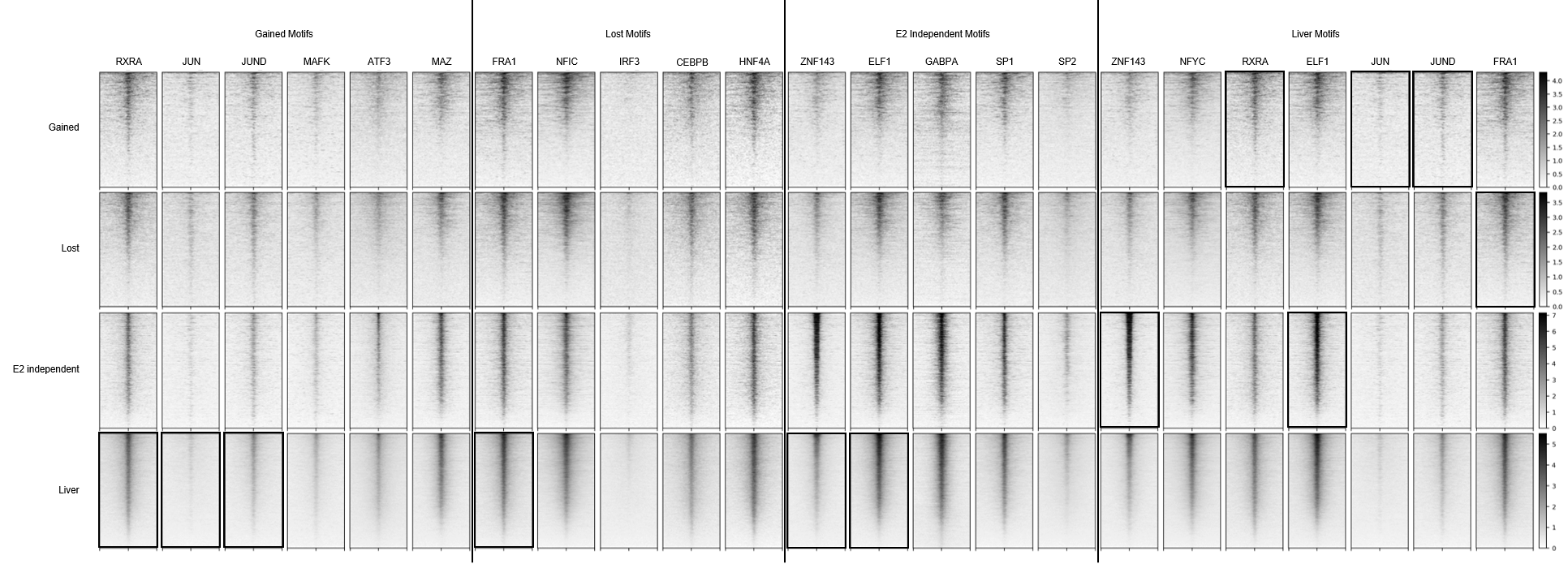

Supplement: Supplementary file 1 [file ijms-22-01461-s001.zip › Supplemental_Figure_2.tif]
